# Respiratory Failure With Pregnancy: Acute Exacerbation of Asthma or Cardiogenic Pulmonary Oedema

**DOI:** 10.7759/cureus.93527

**Published:** 2025-09-29

**Authors:** Megha Gupta, Dhruva Sharma

**Affiliations:** 1 Obstetrics and Gynaecology, The James Cook University Hospital, Middlesbrough, GBR; 2 Obstetrics and Gynaecology, Northampton General Hospital, Northampton, GBR; 3 Critical Care Medicine, The James Cook University Hospital, Middlesbrough, GBR

**Keywords:** balloon mitral valvotomy, echocardiogram, mechanical ventilation, missed miscarriage, mitral stenosis, respiratory failure

## Abstract

We present a complex case involving a 37-year-old pregnant woman at 13 weeks of gestation who developed respiratory failure. The patient had a known history of asthma, which initially created a diagnostic challenge, resulting in treatment for an acute asthma exacerbation. However, as the patient’s clinical condition deteriorated, further investigations were conducted, leading to a diagnosis of severe rheumatic mitral valve stenosis. The patient underwent emergency mitral valve replacement, unfortunately resulting in a miscarriage. Nevertheless, the final maternal outcome was favourable.

## Introduction

Approximately 0.1%-0.2% of pregnant patients may experience respiratory failure. This condition can arise from pregnancy-related complications such as preeclampsia, amniotic fluid embolism or peripartum cardiomyopathy, as well as from non-pregnancy-related causes, including exacerbations of asthma, bacterial pneumonia or restrictive lung disease. Additionally, certain pre-existing conditions may worsen during pregnancy, leading to respiratory failure, such as pulmonary oedema secondary to cardiac disease, pulmonary embolism or aspiration pneumonitis. Management strategies during pregnancy are generally consistent with those employed for non-pregnant patients [1].

Breathlessness during pregnancy can result from a multitude of causes. The most prevalent differential diagnoses include physiological changes, anaemia, asthma, pulmonary embolism, mitral stenosis, peripartum cardiomyopathy, pneumonia, pneumothorax and hyperventilation secondary to anxiety [2]. Establishing the underlying cause of respiratory failure through a thorough clinical assessment and diagnostic investigations is essential for ensuring favourable maternal and foetal outcomes.

Pregnancy is associated with significant cardiovascular adaptations, including increased heart rate and cardiac output, which may exacerbate pre-existing cardiac conditions or uncover previously undiagnosed cardiac diseases [3]. These physiological changes can commence as early as eight weeks of gestation, with cardiac output increasing by approximately 20% [2]. Women with cyanotic heart disease, mitral stenosis, aortic stenosis or systolic dysfunction are particularly vulnerable to cardiac decompensation [4]. Furthermore, the colloid osmotic pressure/pulmonary capillary wedge pressure (PCWP) gradient is reduced by about 30% during pregnancy, elevating the risk of pulmonary oedema in pregnant patients with underlying conditions such as cardiac disease or hypertension [2].

Approximately 1%-4% of all pregnancies are associated with cardiovascular conditions, which account for 26.5% of maternal deaths. Consequently, cardiovascular disease remains one of the leading causes of maternal mortality in developed countries [5,6]. Asthma is among the most common pulmonary disorders during pregnancy, affecting approximately 4%-8% of the general population. About one-third of these patients experience an improvement in their condition during pregnancy, while an equal proportion either remains stable or experiences deterioration [7].

This case report aims to highlight the critical importance of investigating cardiac aetiologies of respiratory failure in pregnant patients with a history of asthma.

## Case presentation

A 37-year-old pregnant woman at 13 weeks of gestation presented to the emergency department of a tertiary hospital with severe respiratory distress. The ambulance crew pre-alerted the hospital, indicating a life-threatening asthma attack, and the patient was taken directly to the resuscitation area. Upon arrival, she was alert, oriented and able to speak in small phrases. The patient reported waking up in the middle of the night with shortness of breath, which she described as resembling a panic attack. She had a known history of asthma managed by her general practitioner and was using a Fostair inhaler up to six times per day. Although she had been hospitalised previously for asthma exacerbations, she had never required intubation or mechanical ventilation. In the preceding month, she had two visits to the accident and emergency department for shortness of breath and was managed as an asthma patient. The patient was a smoker, consuming 20 cigarettes per day, but had reduced her intake to four cigarettes daily during her pregnancy.

On examination, her vital signs were as follows: pulse rate of 138 bpm, blood pressure of 157/100 mmHg, respiratory rate of 40 breaths per minute and oxygen saturation of 94% on 5 L of oxygen. Chest auscultation revealed good air entry bilaterally, with diffuse wheezing and harsh, coarse bilateral basal crackles. On cardiovascular examination, jugular venous pressure was not raised, there was no peripheral oedema and all peripheral pulses were palpable. It was difficult to hear the heart sounds as they were masked by loud respiratory sounds. The patient appeared visibly distressed and was sitting in a tripod position. Initial arterial blood gas analysis was suggestive of hypoxaemia with normocapnia, and the patient was started on treatment with an initial working diagnosis of an acute exacerbation of asthma (Table 1). Her management included back-to-back nebulisations with salbutamol and ipratropium, intravenous magnesium sulphate, intravenous hydrocortisone and aminophylline infusion. Although the patient showed initial mild improvement, her condition deteriorated over the next few hours. Oxygen saturation declined, and CO2 levels began to rise in subsequent blood gas analyses. Due to worsening arterial blood gases, she was intubated and required ventilatory support.

**Table 1:** ABG of the patient showing significant worsening and respiratory failure ABG: arterial blood gas, pO2: partial pressure of oxygen, pCO2: partial pressure of carbon dioxide

	pH	pO2 (kPa)	pCO2 (kPa)	Inference
Normal	7.35-7.45	11-13	4.7-6	-
Initial presentation	7.36	10	5.2	Hypoxaemia with normocapnia
On 5 L oxygen	7.18	11.2	8.24	Hypercapnia with acidosis
Post-intubation	7.03	24.4	12.3	Type 2 respiratory failure

**Figure 1:**
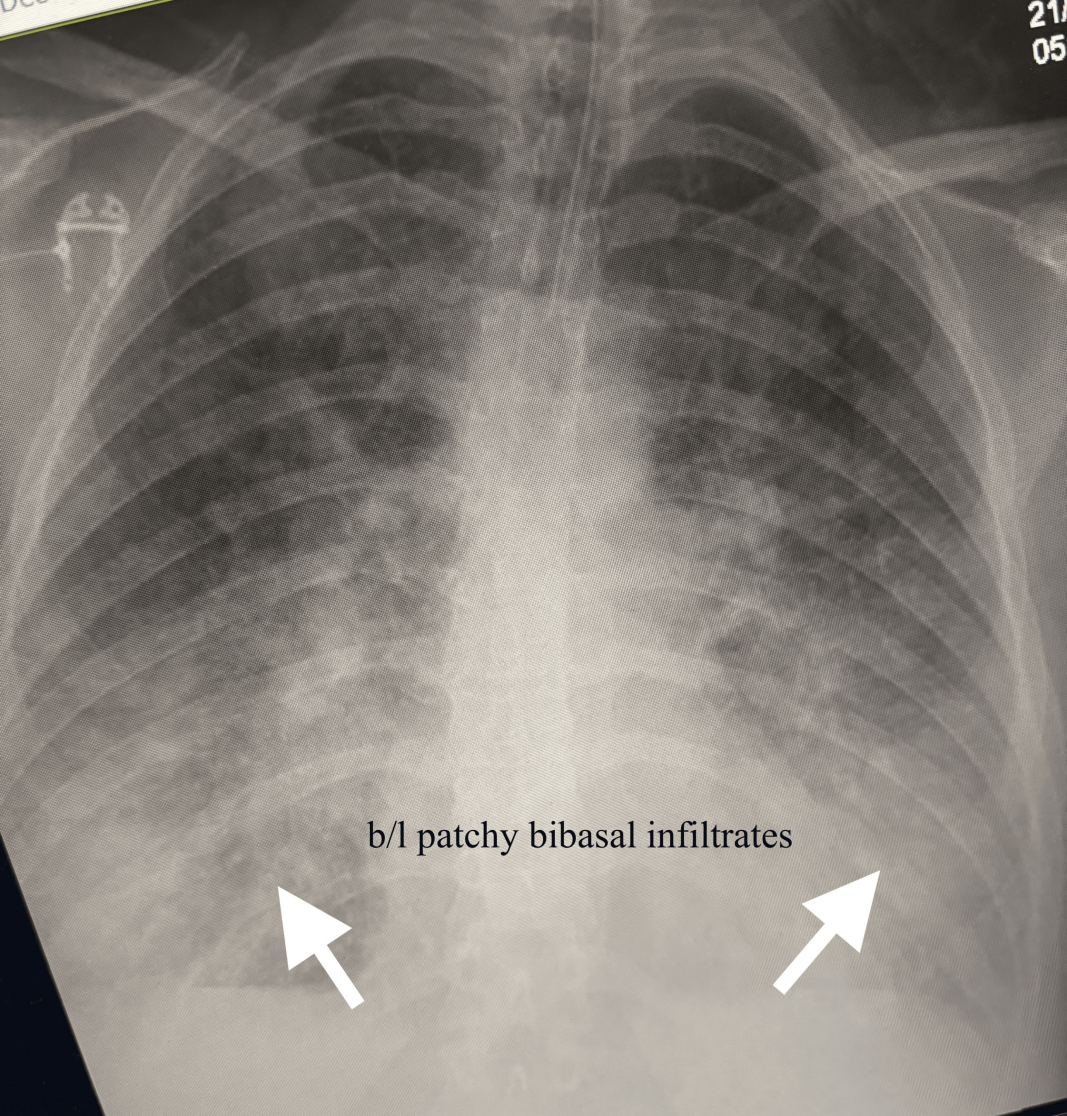
Chest X-ray showing pulmonary oedema

A chest X-ray was done, which showed normal heart size and bilateral patchy bibasal infiltrates, indicating pulmonary oedema, which was further confirmed by frothy secretion from the endotracheal tube (Figure 1). The patient was administered intravenous furosemide for the oedema. ECG was done, which showed sinus tachycardia and no other findings. A bedside echocardiogram demonstrated hyperdynamic left ventricular function with a limited view, suggesting non-cardiogenic pulmonary oedema. A formal echocardiogram was recommended for detailed valve assessment. Findings from the computed tomography pulmonary angiography (CTPA) were consistent with severe pulmonary oedema, with no evidence of pulmonary embolism.

The patient was sedated and ventilated overnight. Attempts to wean her off ventilation were complicated by episodes of desaturation and recurrent pulmonary oedema. As the clinical picture shifted from an asthma exacerbation to suspected pulmonary oedema of unknown origin, the ventilator settings were adjusted accordingly. A subsequent formal echocardiogram suggested severe mitral stenosis, likely related to rheumatic heart disease (RHD), with a mitral valve pressure gradient of 28 mmHg. Unfortunately, mitral valve planimetry was not available. The left ventricle was normal in size. Ultimately, a final diagnosis of cardiogenic pulmonary oedema secondary to critical rheumatic mitral stenosis was established. The obstetric team advised that treatment should proceed as per the medical team’s recommendations. A referral to cardiothoracic surgery was made, and the patient was scheduled for balloon valvotomy due to life-threatening pulmonary oedema requiring mechanical ventilation. However, the procedure was complicated by severe mitral regurgitation, necessitating emergency valve replacement surgery with a tissue valve. The patient subsequently improved and was transitioned to the high dependency unit (HDU).

Regrettably, the patient experienced a missed miscarriage, which was followed by a semi-elective evacuation of retained products of conception. She was evaluated by the cardiac rehabilitation team six weeks later and was reported to be doing well.

The patient was advised to use contraception by the obstetric team, and the need for preconceptional counselling was emphasised to her.

## Discussion

Rheumatic heart disease (RHD) affects approximately 1% of the global population and represents the most common acquired heart condition among women of reproductive age [8,9]. This pathology is primarily a sequela of rheumatic fever, with mitral stenosis accounting for 90% of RHD cases during pregnancy [2]. It is imperative to evaluate cardiac aetiologies in any pregnant patient presenting with respiratory failure, as RHD may manifest for the first time in this context. Notably, a study indicated that 66.5% of diagnoses of RHD occurred during pregnancy [10]. Failure to recognise conditions such as mitral stenosis can result in life-threatening complications.

In the present case report, the patient had a history of multiple admissions for dyspnoea, receiving a diagnosis of asthma each time. The clinical manifestations of asthma can overlap with those of cardiogenic pulmonary oedema, complicating the differential diagnosis, as both may present with wheezing. Misdiagnosis and inappropriate treatment with beta-2 agonists can exacerbate the patient’s clinical state. The patient also exhibited classical symptoms of paroxysmal nocturnal dyspnoea (PND) upon initial presentation and showed no improvement despite repeated nebulisation with bronchodilators, further raising suspicion for an alternative aetiology of pulmonary oedema beyond asthma exacerbation (Table 2).

**Table 2:** Differentiating features of exacerbation of asthma from cardiogenic pulmonary oedema in this patient JVP: jugular venous pressure, ABG: arterial blood gas, IV: intravenous

	Exacerbation of asthma	Cardiogenic pulmonary oedema
Clinical signs and symptoms	Known asthmatic and smoker, recent recurrent attacks of asthma, bilateral wheezing on chest auscultation, no peripheral oedema, normal JVP, no history of cardiac disease	Clear history of paroxysmal nocturnal dyspnoea at presentation, bilateral basal crept on auscultation suggestive of pulmonary oedema, increased cardiac output in pregnancy can uncover a previous undiagnosed cardiac condition
Investigation	Initial ABG showed normal pH and near-normal ventilation	Worsening ABG suggestive of respiratory failure and acidosis, chest X-ray suggestive of pulmonary oedema, echo suggestive of severe mitral stenosis
Treatment response	Patient showed initial response to bronchodilator therapy	Worsening ABG despite repetitive nebulisation with bronchodilators, aminophylline infusion, IV magnesium sulphate and IV hydrocortisone therapy

In this instance, life-threatening pulmonary oedema was attributed to severe mitral stenosis, diagnosed for the first time in the setting of pregnancy. The physiological changes of pregnancy can precipitate rapid deterioration and the onset of pulmonary oedema in women with critical mitral stenosis, with tachycardia being a significant precipitating factor. This condition exacerbates diastolic filling impairment, resulting in elevated left atrial pressure and subsequent pulmonary oedema [2].

One of the limitations of this case report is the absence of a classical mid-diastolic murmur seen in critical mitral stenosis. This can be due to masking of heart sounds by loud respiratory sounds, as mentioned in the case report earlier. Another reason is that the patient went to florid pulmonary oedema soon after intubation for ventilatory support, masking the murmur further.

Critical investigations to elucidate the underlying cause include chest X-ray, echocardiography and computed tomography pulmonary angiography (CTPA). Severe mitral stenosis is associated with poor foetal outcomes, as in this patient. Data indicate that four out of six women with severe mitral stenosis experienced miscarriage [11]. Furthermore, severe mitral stenosis is classified as World Health Organization (WHO) risk category 4 for cardiovascular disease, indicating that pregnancy is contraindicated due to the high risk of maternal morbidity and mortality, which can range from 40% to 100% [5].

Management of patients with severe mitral stenosis typically involves percutaneous balloon mitral valvuloplasty, ideally performed during the second trimester to minimise foetal risks. Studies suggest that approximately one-fourth of patients with severe mitral stenosis may require surgical intervention in the form of balloon valvotomy during pregnancy [12,13].

In this case, the patient was initially managed with balloon valvotomy but subsequently required valve replacement due to complications from mitral regurgitation, opting for a bioprosthetic valve. The decision between a mechanical valve and a bioprosthetic valve during pregnancy poses significant challenges. Given that pregnancy is a thrombogenic state, women with mechanical valves necessitate lifelong anticoagulation, which carries risks of bleeding and teratogenicity. Conversely, while bioprosthetic valves do not require anticoagulation, they have a limited lifespan, necessitating careful consideration of the long-term management strategy in this patient population [14].

## Conclusions

This case report highlights the clinical symptoms, signs, investigation results and treatment response that can assist in distinguishing an acute asthma attack from cardiogenic pulmonary oedema. Respiratory failure is relatively rare in pregnancy but presents significant risks for both the mother and the foetus, as seen in this case report, where the patient had an adverse foetal outcome in the form of miscarriage. The causes of respiratory failure can be pregnancy-related, cardiac or pulmonary in nature. Clinicians must maintain a high index of suspicion for cardiac aetiologies, as respiratory failure can represent the initial manifestation of rheumatic heart disease during pregnancy, even in patients with a history of asthma, as seen in this case report. Effective management requires a multidisciplinary approach, involving specialists from obstetrics, critical care, cardiology, pulmonology and cardiothoracic surgery, alongside pertinent investigations and comprehensive clinical assessments. Severe mitral stenosis is associated with a high maternal mortality rate, making it essential to provide women with preconception counselling and appropriate management strategies prior to pregnancy.
